# p73 promotes glioblastoma cell invasion by directly activating POSTN (periostin) expression

**DOI:** 10.18632/oncotarget.7600

**Published:** 2016-02-22

**Authors:** Vivien Landré, Alexey Antonov, Richard Knight, Gerry Melino

**Affiliations:** ^1^ Medical Research Council Toxicology Unit, Leicester LE1 9HN, UK; ^2^ University of Rome Tor Vergata, 00133-Rome, Italy

**Keywords:** periostin, p53 family, cell death, temozolomide, metastasis

## Abstract

Glioblastoma Multiforme is one of the most highly metastatic cancers and constitutes 70% of all gliomas. Despite aggressive treatments these tumours have an exceptionally bad prognosis, mainly due to therapy resistance and tumour recurrence. Here we show that the transcription factor p73 confers an invasive phenotype by directly activating expression of POSTN (periostin, HGNC:16953) in glioblastoma cells. Knock down of endogenous p73 reduces invasiveness and chemo-resistance, and promotes differentiation *in vitro.* Using chromatin immunoprecipitation and reporter assays we demonstrate that POSTN, an integrin binding protein that has recently been shown to play a major role in metastasis, is a transcriptional target of TAp73. We further show that POSTN overexpression is sufficient to rescue the invasive phenotype of glioblastoma cells after p73 knock down. Additionally, bioinformatics analysis revealed that an intact p73/POSTN axis, where POSTN and p73 expression is correlated, predicts bad prognosis in several cancer types. Taken together, our results support a novel role of TAp73 in controlling glioblastoma cell invasion by regulating the expression of the matricellular protein POSTN.

## INTRODUCTION

Glioblastoma Multiforme (GBM) is the most common malignant brain tumour in adult patients with an extremely poor prognosis of only 15 month median survival after diagnosis [1]. Despite aggressive treatment, usually consisting of tumour resection followed by radiation- and chemotherapy, tumour recurrence and therapeutic resistance is exceptionally common, making these tumours de facto incurable [2–6].

Several studies over the last decade have suggested that GBM develops from a subsection of tumour cells called stem-like glioblastoma cells [7–9]. These cells, characterised by a self-renewing, often chemo-resistant and immature differentiation phenotype, have tumour-initiating properties and are largely believed to be responsible for tumour recurrence and therapeutic resistance. Even single cells that survive therapy have the ability to initiate growth of a new tumour, and are therefore also called tumour-seeding cells [2, 10]. This suggests that differentiation therapies that induce cancer stem-cell differentiation would be a promising approach to target GBM [11–13]. While differentiation therapy is successfully used to treat acute promyeloctic leukaemia, where it leads to a dramatic increase of patient survival [14], no drug that induces differentiation in solid tumours has yet been developed. This is mainly due to a lack of development-based classification of these tumours, which often have considerably more varied genetic abnormalities compared to acute promyeloctic leukaemia, as well as our limited understanding of the pathways and components involved in the differentiation of different cancer types [14].

The transcription factor p73 is a member of the p53 family, which comprises three members p53, p63 and p73 that share similarities in their structure and function [15–19]. Having been discovered already in 1979, p53 is the most studied component of this family of transcription factors, showing a very complex gene activation program spanning from autophagy [20, 21], ROS, metabolism and mitochondria regulation [22–24], DNA damage response [25–29] to stemness and lineage determination [30–32]. Despite 35 years of high quality research, several crucial issues remain unanswered in order to fully understand the biological role and function of this tumour suppressor. In fact p53, as well as its family members, shows an extreme complexity, including, for example, its stability and degradation [33–37], its connection and regulation to micro-RNA [38–44] and its splicing isoforms [45, 46]. In parallel to the advances in p53 biology, crucial progress is also under way for its therapeutic exploitation [47–56]. Although having been identified much later, p63 and p73 already show their complexity and interaction with p53 [57–63]; where p63 function is highly relevant in skin formation and homeostasis [64] as well as in cancer [63, 65, 66]. p73 exists in a variety of isoforms which can be divided into transactivation (TA) domain containing isoforms (TAp73) and those lacking the TA domain (ΔNp73), and each of these can be expressed as a number of C-terminal isoforms to exert distinct functions [67–74]. Depending on cell type and p73 isoform, the protein has been shown to exhibit both tumour suppressive and oncogenic functions [75–77]. Unlike p53, p73 is rarely mutated in human cancers and both the TA and ΔN isoforms have been shown to be overexpressed in several tumour types [78, 79]. p73 deficient mice have striking developmental deficiencies in the CNS, mainly characterised by cortical loss leading to ex vacuo hydrocephalus as well as dysgenesis of the hippocampus and caudal cortex [80, 81]. Interestingly, these severe neurological deficiencies are phenocopied neither by the ΔNp73 nor the TAp73 KO mice, suggesting redundancy of the two isoforms during neurogenesis [82]. TAp73 KO mice display several abnormalities in the hippocampus, while ΔNp73 KO mice are healthy and show a relatively mild neurological phenotype with loss of the hydrocephalus, but no cortical loss [83–85].

Here we demonstrate that p73 plays a role in glioblastoma cell differentiation, since loss of p73 in glioblastoma cells leads to a more differentiated phenotype with reduced migration and invasion abilities. Furthermore, we show that the extracellular protein POSTN (periostin, osteoblast specific factor, HGNC:16953), that has been shown to play a role in glioblastoma carcinogenesis and is an important factor in metastasis, is transcriptionally activated by p73, and that this activation leads to increased cell invasion of glioblastoma cells *in vitro*.

## RESULTS

### Loss of p73 leads to glioblastoma cell differentiation

**Figure 1:**
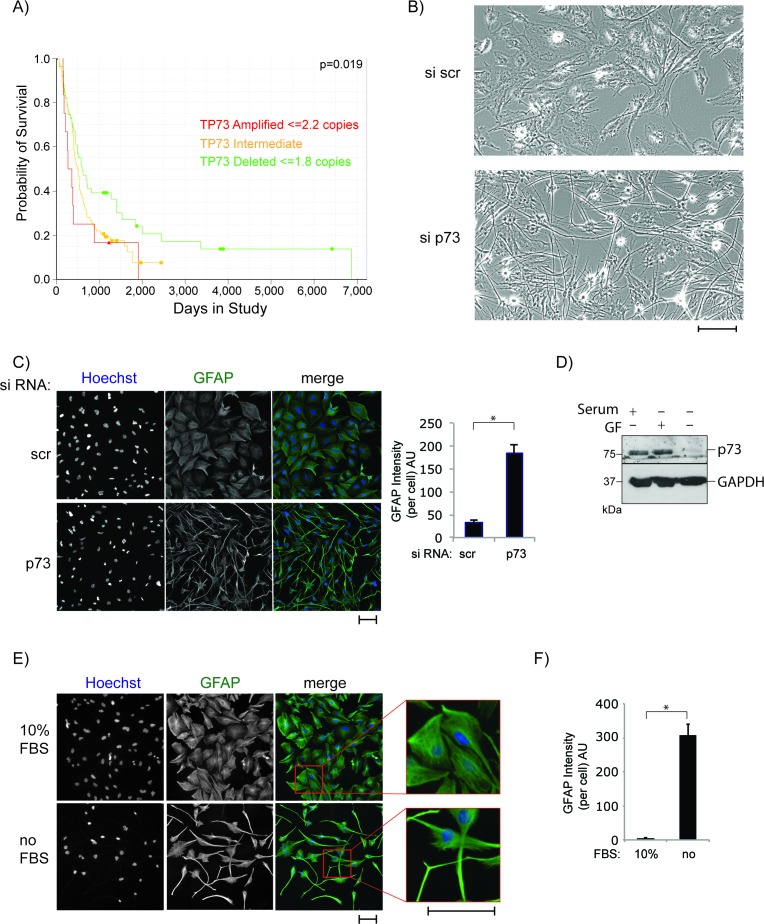
P73 knock down induces morphological transformation of glioblastoma cells **A.** p73 gene deletion is correlated with prolonged survival of glioblastoma patients. Data is from the REMBRANT database/NIH (https://caintegrator.nci.nih.gov/rembrandt/). The *p*-value corresponds to the gene deletion group in comparison to all other patients. **B.** Morphological changes of U251 cells after 72 h of p73 knock down using siRNA transfection. **C.** Cells as in B but fixed and stained with an anti-GFAP antibody. Pictures were taken using the Cellomics, GFAP signal was quantified and is expressed as arbitrary units (right panel). **D.** Cells were grown in media that was serum rich (10%), serum free or media complemented with growth factors (GF) (20 ng/ml EGF and FGF) and p27 supplement. Total protein was extracted and blotted with antibodies against p73 and GAPDH. **E.** Cells were incubated in serum free medium or full medium for 72 h and visualised and quantified (F) using a GFAP antibody as in C. Scale Bars in B, C and E represent 100 μm. *p < 0.0001. Error bars represent SEM.

Although the role of p73 in neuronal development and differentiation has been studied extensively in recent years, relatively little attention has been paid to its role in the development and metastasis of malignancies originating in the brain. Several studies in GBM [86–88] revealed a correlation between mRNA levels of the TAp73 and ΔNp73 isoforms and tumour grade as well as patient survival, leading to the suggestion that p73 may be a potential biomarker of GBM tumour grade. Furthermore, analysis of datasets of glioma patients showed a correlation between p73 gene deletion and longer survival (Figure 1A). Therefore, as a first step to understand the potential mechanism for this correlation, we knocked down total p73 mRNA using siRNA in the U251 and U87 glioblastoma cell lines (Figures 1B, S1, S2A). Strikingly, p73 knock down resulted in a dramatic change of cell morphology, from a flat polygonal shape to a small, round cell body with very long and fine processes, an appearance, which resembles that of mature astrocytes. To further demonstrate that the change in morphology is due to cell differentiation, glioblastoma cells, transfected with control siRNA or siRNA specific for total p73, were stained using a GFAP (glial fibrillary acidic protein) antibody (Figures 1C, S2B). GFAP is an intermediate filament that is highly expressed in differentiated astrocytes. The GFAP signal in the cell processes was quantified, and the results showed that GFAP expression is higher in the cells in which p73 had been knocked down (Figure 1C, right panel). This observation suggests that glioblastoma cells may differentiate into astrocytes in the absence of p73. This is also in line with previous studies published by our group showing a decrease in stemness markers in neurons isolated from p73 KO compared to wt mice [89] and reduced Nestin expression, a neuronal stem cell marker, in the dentate gyrus of p73 KO mice [90].

As these results suggest a role of p73 in regulating the differentiation status of glioblastoma cells, we were interested in the effect of induced differentiation on p73 protein levels. To study this, differentiation of glioblastoma cells was induced by serum withdrawal (Figure 1E, 1F) and p73 levels were determined by western blotting (Figure 1D). Serum withdrawal led to almost complete loss of p73 protein expression, while no change in p73 protein levels was seen when serum was replaced by growth factors and supplements promoting stem-cell-like properties [91].

### p73 regulates cell migration and invasion

**Figure 2:**
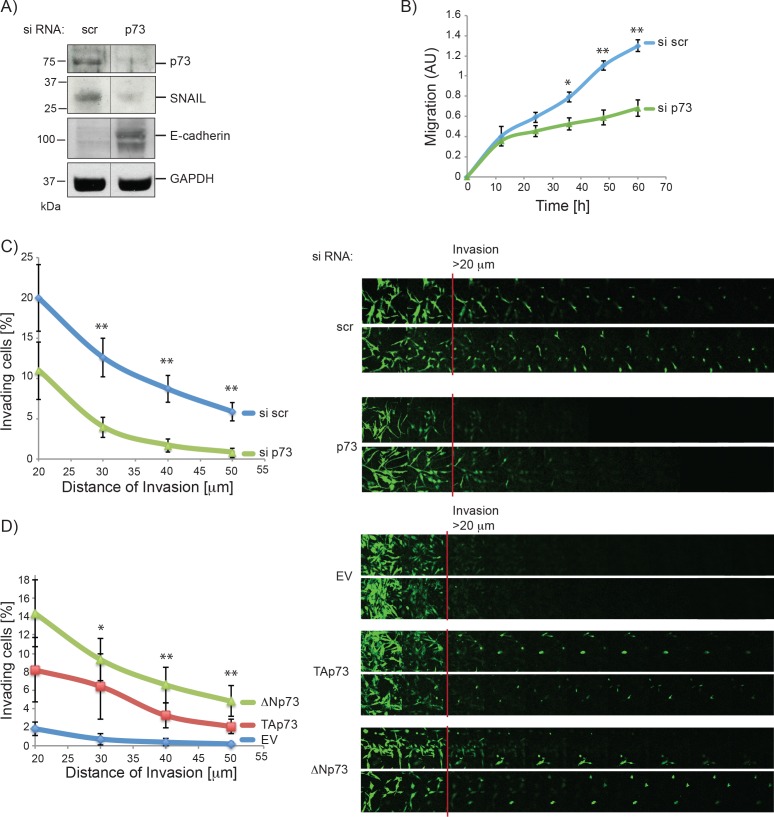
P73 knock down reduces migration and invasion of glioblastoma cells **A.** Whole protein extract of U251 cells 72 h post-transfection with scr or p73 siRNA was analysed by immunoblotting with antibodies against p73, SNAIL, E-cadherin and GAPDH. Line indicates where 2 lanes that are not next to each other on the gel were moved side by side (see Figure S3A for full scan). **B.** The migration ability of U251 cells after siRNA transfection (scr or p73) was determined using the xCelligence. **C.** Invasion into matrigel of U251 cells that were transfected with siRNAs targeting p73 or a non-targeting control is shown. **D.** As in C but cells were transiently transfected with plasmids encoding TAp73α, ΔNp73α or empty vector control. *P*-values shown are for EV compared to TAp73 and EV compared ΔNp73. *p < 0.1, **p < 0.05. Error bars represent SEM.

The fact that stem-like glioblastoma cells are generally associated with a mesenchymal phenotype, and that p73 affects the differentiation status of glioblastoma cells, suggests that p73 can affect epithelial to mesenchymal transition (EMT). We therefore performed western blot analysis of different EMT markers after total p73 knock down. As shown in Figure 2A (and S3A), transient knock down of p73 led to a remarkable reduction of SNAIL, an important factor in the delamination process in neuronal tissue development, together with up-regulation of E-cadherin [92, 93]. We observed no change in other EMT marker i.e. Twist and Vimentin (data not shown). EMT is generally associated with an invasive phenotype, and we therefore wanted to establish whether p73 affects the migration and/or invasion ability of glioblastoma cells. Cell migration was assayed using the xCELLigence system, whereby cells that migrated through a membrane, from serum free towards serum containing media, were quantified. The assay showed a striking reduction in cell migration ability after knock down of p73 (Figure 2B). Similarly, in an invasion assay, where the ability of the cells to enter and invade a matrigel matrix was assessed, knock down of total p73 resulted in consistent reduction of invasion (Figure 2C), whereas overexpression of p73 isoforms led to an increase in matrigel invasion (Figures 2D, S3B). Together these results demonstrate a role of p73 in glioblastoma cell morphology, associated with a more invasive phenotype.

**Figure 3:**
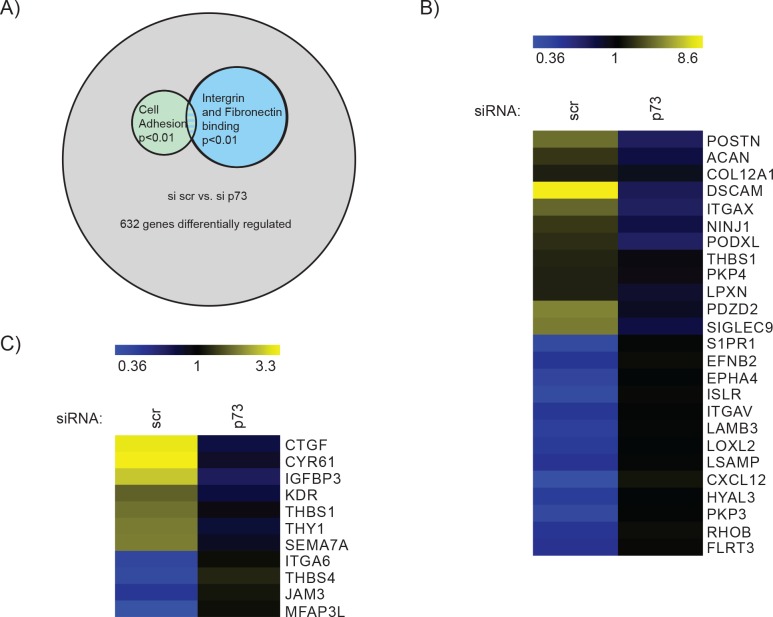
Analysis of mRNA expression after p73 knock down suggests a role for p73 in cell migration and invasion **A.** Results of the microarray revealed 632 genes that were differentially expressed in control cells compared to knock down of endogenous p73 (Schematic illustration of gene array results). Pathway analysis of microarray results comparing U251 transfected with scr siRNA and p73 siRNA indicated changes in the pathways shown. Odds ratios for pathways are Integrin binding = 4.4, Fibronectin binding 9.05 and Cell adhesion = 2.04. p- values refer to the significance of enrichment of genes of the pathways shown. Heat maps of **B.** “Integrin and Fibronectin binding” (combined), 10 genes for integrin binding and 5 genes for fibronectin binding were identified, while CTGF is involved in both pathways. **C.** “Cell Adhesion” genes identified by GO term analysis, as differentially expressed between si scr and si p73.

To gain insight into the molecular mechanisms underlying these changes of U251 morphology and invasion, we performed a gene microarray analysis of U251 cells transfected with siRNA for total p73 or a scrambled sequence for 72 h. We found 632 genes differentially expressed in the knock down compared to the control cells (Figure 3A). Using a pathway analysis tool [94], we identified the pathways most enriched in differentially expressed genes to be: Integrin binding, fibronectin binding, blood coagulation and cell adhesion (Figure 3A–3C). This molecular data is in agreement with the biological effect of p73 in cell migration, as integrin and fibronectin as well as cell adhesion pathways are all involved in cell mobility, and alterations of these pathways have been shown to be involved in metastasis of cancer cells [95]. To verify the results of the microarray we tested the expression of the top ten down- and seven up-regulated genes and found that we could validate ~ 73 % of the genes in the array (Figure S4).

### POSTN is a direct target of p73

**Figure 4:**
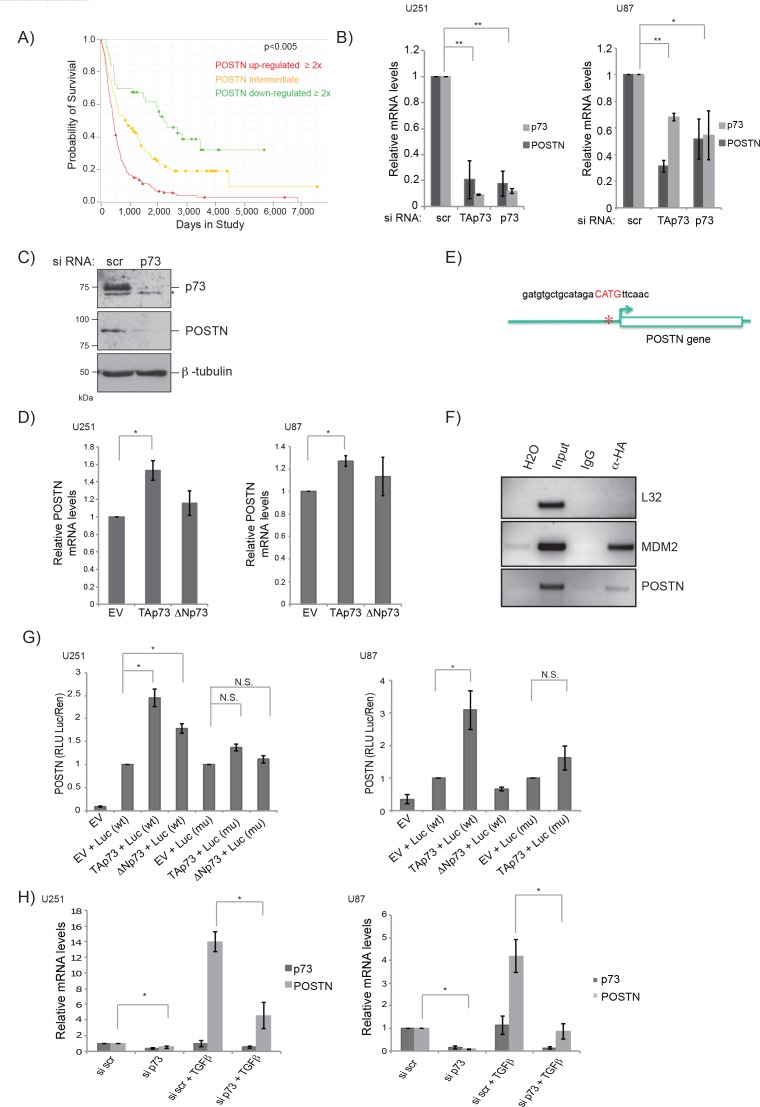
POSTN is a direct target of p73 **A.** High POSTN mRNA expression is strongly correlated with reduced survival of glioblastoma patients. Data is from the REMBRANT database/NIH (https://caintegrator.nci.nih.gov/rembrandt/). *p*-value corresponds to comparison between all three conditions with each other. **B.** Expression of POSTN mRNA 72 h after transfection with p73 or scr siRNA was determined using RT- qPCR in U251 (left panel) and U87 (right panel) cells. **C.** U251 protein extracts of cells transfected with siRNA for p73 or a scrambled control and analysed by immunoblotting using p73, POSTN and β-tubulin antibodies. Asterisk indicates an unspecific band of the p73 antibody. **D.** Expression of POSTN mRNA 24 h after transfection with plasmids encoding TAp73α, ΔNp73α or an empty vector control was determined using RT-qPCR in U251 (left panel) and U87 (right panel) cells. **E.** Analysis of the POSTN promoter revealed one potential binding site for transcription factors of the p53 family 600 bp upstream of the transcriptional start site. **F.** ChIP assay demonstrating binding of TAp73α to the POSTN promoter in U251 cells. **G.** U251 (left panel) or U87 (right panel) cells were transfected with p73 constructs (TAp73α or ΔNp73α) plus POSTN-Luc wt or mutant and control Renilla-Luc. Post transfection (24 h), cells were harvested and dual luciferase reporter assays performed. Results were normalised by expressing firefly/renilla luciferase activity in relative light units (RLU) as the mean +/− S.D.H. **H.** U251 (left panel) or U87 (right panel) cells were transfected with siRNA (p73 or scrambled control), 48 h post transfection cells were treated with 5 ng/ml TGFβ for another 24 h. Cells were harvested and mRNA expression of p73 and POSTN was analysed using RT-qPCR. *p < 0.05, **p < 0.001. Error bars represent SEM.

To gain insight into the mechanism of how p73 regulates cell migration, we decided to study the regulation of one of the genes identified in the microarray in more detail. We chose POSTN (Periostin), as it has previously been implicated in glioblastoma malignancy and to be important for EMT and cancer metastasis [96–98]. The matricellular protein POSTN was initially identified as a cell adhesion protein in a mouse osteoblastic cell line [99, 100]. More recently a role of POSTN in carcinogenesis and metastasis has been discovered [101, 102]. Numerous studies showed an up-regulation of both POSTN protein and mRNA levels in a plethora of different tumours, including glioblastoma, neuroblastoma, breast, colon and pancreatic cancer [96, 101]. We used the Rembrandt database to examine the effect of POSTN mRNA up-regulation on patient survival and found that POSTN overexpression correlates strongly with poor prognosis in glioblastoma patients (Figure 4A). To study the regulation of POSTN by p73, we initially used knock down experiments in U251 and U87 glioblastoma cell lines, where both full p73 and specifically the transcriptionally active TAp73 isoform expression were abolished. As shown in Figure 4B, both the isoform specific and total knock down resulted in a striking decrease of POSTN mRNA levels in both cell lines. Furthermore, knock down of p73 also led to a marked decrease of POSTN protein levels (Figure 4C). Correspondingly, when TAp73 is overexpressed there is a small, but significant increase in POSTN mRNA levels, while ΔNp73 has no effect on its expression (Figure 4D).

To investigate whether POSTN is a direct transcriptional target of TAp73 we performed a promoter analysis of the POSTN gene and found one potential p73 binding site ~600 bp upstream of the POSTN transcriptional start site (Figure 4E). We employed chromatin immunoprecipitation (ChIP) assays to investigate binding of TAp73 to this potential binding site using overexpression of TAp73α-HA in U251 cells followed by DNA-protein crosslinking and isolation of TAp73α using an HA antibody. The results showed that TAp73 bound to the POSTN promoter, as well as to the MDM2 promoter used as a positive control (Figure 4F). To confirm that TAp73 activates the POSTN promoter, reporter assays were carried out using the POSTN promoter, either in its wild type form or with a three-nucleotide deletion in the previously identified p73 binding site (Figure 4G). The results showed that TAp73, and in U251 cells also ΔNp73, transcriptionally activates reporter activity in both U251 and U87 cells, and that this is abrogated when the p73 binding site is mutated. Taken together the data from the ChIP and reporter assays demonstrated direct binding of TAp73 to the POSTN promoter leading to POSTN transcription.

Expression of POSTN is induced via the TGFβ pathway, and we next asked whether p73 is also required to activate POSTN expression when induced by TGFβ. To test this, cells were transfected with either siRNA targeting total p73 or a scrambled control, treated with TGFβ and mRNA levels of POSTN and p73 were measured using RT-qPCR. As expected, TGFβ treatment led to a strong increase in POSTN mRNA and knock down of p73 reduced this by around 3 fold, further demonstrating that p73 plays a role in POSTN activation (Figure 4H). The fact that p73 knock down reduced the activation of POSTN in response to TGFβ treatment suggests that p73 acts downstream of TGFβ in this pathway. It remains to be investigated how/if p73 is directly activated by TGFβ leading to an increase of POSTN expression. p73 mRNA levels do not change after TGFβ treatment, suggesting that p73 activity could be modified by post translational modifications.

**Figure 5:**
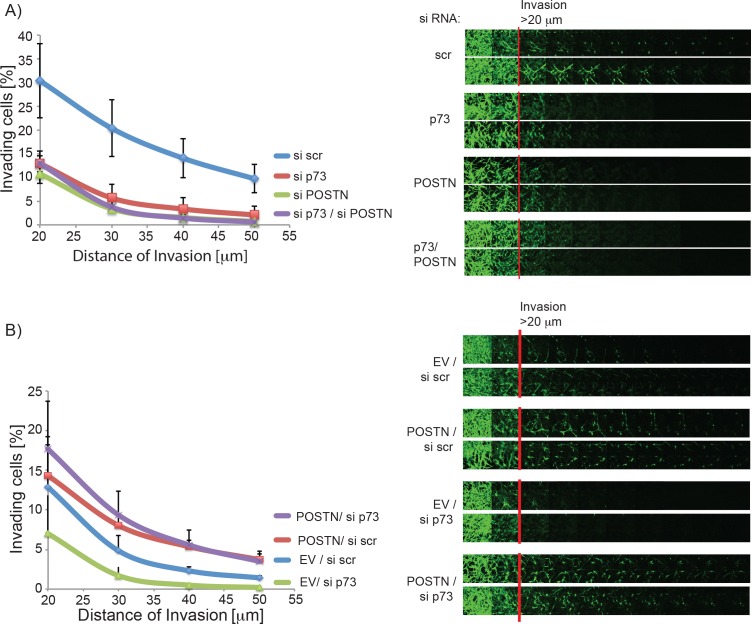
POSTN overexpression rescues invasion ability of glioblastoma cells with p73 knock down **A.** Invasion into matrigel of U251 cells that were transfected with siRNA targeting p73, POSTN, both or a nontargeting control is shown. **B**. As in A, but cells were transfected with siRNA (scr or p73) and plasmids encoding POSTN or empty vector as indicated.

Since POSTN is known to promote invasion of glioblastoma cells we speculated that the reduction of POSTN after p73 knock down is, at least in part, responsible for the loss of invasive ability of the cells. To test this we performed an invasion assay with cells transfected with siRNA for total p73, POSTN or both. As expected loss of either p73 or POSTN decreased invasion of glioblastoma cells; however, a double knock down of both did not lead to further reduction in invasion, suggesting that indeed both proteins are acting in the same pathway with p73 regulating POSTN expression (Figures 5A, S5A). To further support this hypothesis, we overexpressed POSTN in cells (Figure S5B) transfected with either siRNA for total p73 or control cells to see if POSTN expression can rescue the invasive phenotype of these cells. As shown in Figure 5B, overexpression of POSTN led to a strong increase in invasion that is not reduced after p73 knock down. Moreover, the reduced invasion produced by p73 knock down is rescued by overexpression of POSTN.

Taken together this data demonstrates that p73 regulates POSTN levels and thereby invasion of glioblastoma cells.

### Loss of p73 leads to a decreased chemo-resistance

**Figure 6:**
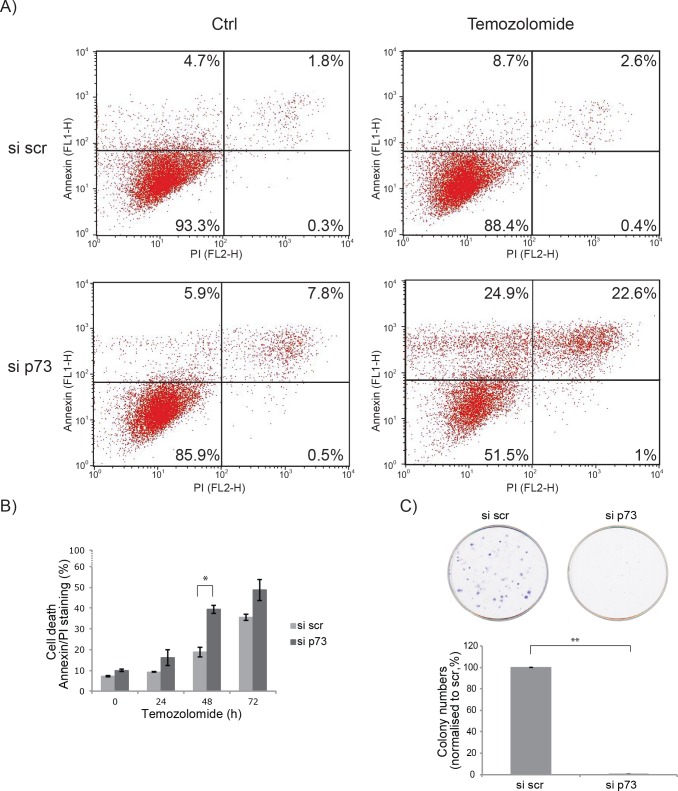
Glioblastoma are more chemo-sensitive after p73 knock down **A.** U251 cells were treated with 50 μM Temozolomide for 48 h. Early and late apoptosis was detected using Annexin V/PI-double staining followed by flow cytometry analysis. **B.** As in A, but with additional time points of Temozolomide treatment of 24 and 72 h, total apoptosis was quantified and is shown. **C.** Colony formation assay after p73 knock down. *p = 0.05, **p < 0.0001. Error bars represent SEM.

As an undifferentiated phenotype of glioblastoma cells is usually associated with increased invasiveness and chemoresistance, we investigated the effect of total p73 knock down on glioblastoma cell sensitivity to Temozolomide. We measured apoptosis in cells after drug treatment using Annexin V/PI staining. p73 knock down led to a modest, but significant, increase of apoptosis after treatment with the Temozolomide (Figure 6A, 6B). Additionally, we examined the effect of POSTN overexpression on apoptosis after Temozolomide treatment with and without p73 knock down. However, no change in cell death after POSTN overexpression was observed, irrespectively of p73 knock down (Figure S6). This suggests that the effect on chemo-resistance after p73 knock down is mediated by a different axis compared to the effect on cell invasion and is POSTN independent.

As we observed cell differentiation after p73 knock down and this is often associated with a decrease in proliferation we, furthermore, probed the effect of p73 on cell proliferation using a colony formation assay. The results showed a dramatic decrease of cell proliferation after total p73 knock down (Figure 6C).

### Correlation between p73 and POSTN levels predicts bad prognosis for patient survival

**Figure 7:**
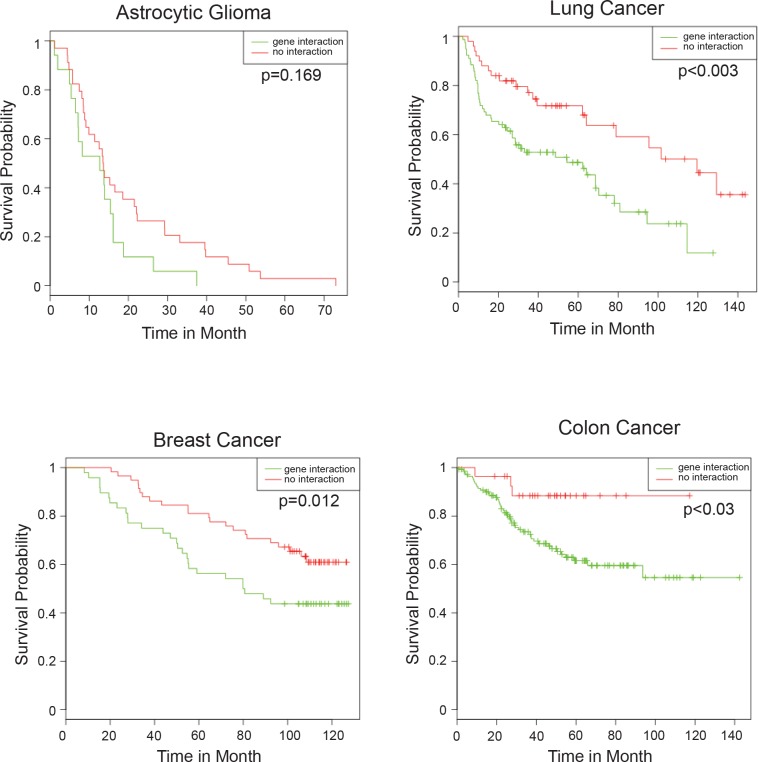
Correlation of p73 and POSTN expression predicts bad patient prognosis Positive total p73/POSTN correlation represents a negative prognostic factor for patient survival in astrocytic gliomas (dataset: GSE18166, *N* = 51), lung (GSE4573, *N* = 128), breast cancer (GSE19783, *N* = 106) and colon cancer (GSE17538, *N* = 566). Panels represent patient survival estimation of p73/POSTN-positive correlation groups compared with negative or absent correlation groups.

If up-regulation of POSTN by p73 leads to increased invasion, we would expect that the tumours in which this regulation is active and thus POSTN and p73 levels correlate, are more aggressive and have a worse prognosis than tumours where this axis is not functional. To assess this bioinformatically, glioblastoma datasets from patients were divided into two groups; those where p73 and POSTN mRNA correlate and those where there is no correlation. We then compared the survival probability of the two groups (Figure 7). While the survival of the group where POSTN and p73 levels correlate is decreased, the difference between this group and that in which there is no interaction does not reach significance, possibly because the sample size is too small. We therefore decided to probe other cancers, specifically lung, breast cancer and colon cancer, where larger datasets are available. And importantly, POSTN was implicated to be a negative prognostic marker in breast [103, 104], lung cancer [105, 106] and colon cancer [107, 108]. All three datasets show a dramatic, and statistically significant, reduction in survival of patients where levels of p73 and POSTN correlate (Figure 7). Taken together we have shown that p73 ablation leads to morphological changes associated with a differentiated phenotype in glioblastoma cells, that leads to a decrease in their invasion ability through inactivation of the p73/POSTN axis.

## DISCUSSION

In the current study we demonstrate that p73 promotes invasion and migration in glioblastoma cells through directly activating expression of POSTN. Previous studies have proposed a role for p73 in cell migration and invasion. Sablina *et al* (2003) showed that TAp73 leads to increased colon cancer cell migration [109], while Zhang *et al* (2012) observed a reduction of cell migration in non-cancerous breast cells (MCF10a) after TAp73 overexpression [110], suggesting that the effect is cell type and condition dependent. The ΔNp73 isoform has also been implicated with increased invasion and metastasis in a study by Steder *et al*, [111], that showed that ΔNp73 can initiate metastasis by inhibiting IGF1R-AKT/STAT3 signalling.

Here we show that, strikingly, loss of p73 leads to a differentiated phenotype that is less invasive and more susceptible to chemotherapeutic treatment in glioblastoma cells. Using a gene microarray, we identified POSTN as a direct transcriptional target of p73 that is strongly down regulated in glioblastoma cells upon p73 loss. A previous study has shown regulation of POSTN mRNA by p73 in a thyroid cancer cell line [109]. Here, however, we show for the first time that p73 directly binds to the POSTN promoter to activate its transcription and that p73 directly regulates POSTN protein levels. The study of POSTN regulation by p73 in thyroid cancer detected a strong upregulation of POSTN expression in a reporter assay in response to ΔNp73 overexpression, while we did not observe an upregulation of POSTN mRNA in response to ΔNp73 and only a mild effect in a reporter assay. This could be due to cell type specific non-transcriptional activation of POSTN by ΔNp73, as this isoforms does not have intrinsic transcriptional activity.

Several studies have demonstrated that POSTN is up regulated in a variety of tumours, including glioblastoma [96, 101], and acts as a metastasis-promoting factor [102]. Likewise, POSTN has been shown to promote epithelial to mesenchymal transition leading to increased cell invasion and proliferation [98, 112, 113]. In addition to being overexpressed by tumour cells, POSTN is also expressed in the stroma of normal stem cell niches and aids the metastatic success of circulating cancer cells [102]. Taken together, these data suggests that POSTN plays an important role in tumour formation and metastasis, being either expressed by the tumour cells or the surrounding stroma.

Here we have demonstrated that TAp73 directly activates POSTN expression leading to an invasive phenotype in glioblastoma cells. Moreover, total p73 knock down renders glioblastoma cells more sensitive to Temozolomide treatment and bioinformatic analysis revealed that correlation of p73 and POSTN expression predicts poor patient survival. This suggests that the p73/POSTN axis is a negative predictive factor of patient survival that might drive carcinogenesis. Further studies, especially *in vivo*, are necessary to establish whether p73 or its target POSTN could provide a drug target for development of new drugs that reduce glioblastoma invasion.

## MATERIALS AND METHODS

### Cell culture, transfections and reporter assay

Glioblastoma cell lines U251 and U87 were maintained in DMEM (Life Technologies) and EMEM (ATCC) respectively; all media were supplemented with 10 % FBS (v/v) (Labtech) and 1 % (v/v) penicillin/streptomycin (Invitrogen), and grown with 5 % CO_2_ at 37°C. To maintain cells, U251 and U87 cells were grown to 90 and 70% confluence respectively and then split 1:8 using trypsin:EDTA (Gibco).

Transfection of cells with siRNA was carried out using Hyperfect (Qiagen) at 60% (U251) and 50% (U87) confluence following the supplier's instructions using 30 nM siRNA, siRNA Silencer Select was from Ambion (IDs: TAp73: 115665, total p73: 2671, POSTN: s20889). DNA transfections were carried out using Effectene (Qiagen) at 80% confluence and Lipofectamine 2000 (Life Technologies) at 70% confluence for U251 and U87 respectively.

### Reporter assay

Reporter activity was tested using the Dual Luciferase Reporter System from Promega. Briefly, cells were seeded in 24 well plates and transfected with Renilla (50 ng/well), POSTN reporter construct wt or mutant (120 ng/well) and 100 ng of the indicated p73 isoforms. After 24 h, cells were washed and lysed on the plate using the Promega lysis buffer. Firefly luciferase and Renilla activities were measured as indicated by Promega. Results shown are firefly luciferase expression normalised by Renilla and are expressed as relative light units (RLU). Results are shown as the median of 3 independent experiments, all carried out in duplicate and measured twice.

### Plasmids, antibodies and western blot

Antibodies were anti-p73 (Melino lab [114]), anti-SNAIL (Cell Signalling L70G2, mAb), anti-E-Cadherin (Cell Signalling 4A2, mAb), anti-POSTN, anti-GFAP (DAKO, pAb). Anti-GAPDH (SIGMA, G8795, mAb) and anti-β-tubulin (Santa Cruz H-235, pAb). Secondary antibodies were HRP conjugated goat-anti-rabbit and goat-anti-mouse (Bio Rad), and Alexa Flour 488 donkey anti-rabbit (Molecular Probes). Cells were lysed in Triton X-100 lysis buffer (50 mM HEPES (pH 8), 0.4 % Triton X-100, 150 mM NaCl, 10 mM NaF, 2 mM DTT, 0.1 mM EDTA, 1x protease inhibitor mix (Roche)). Western Blotting was performed as described previously [115].

The POSTN gene was subcloned into the pcDNA 3.1 (−) vector (Life Technologies) after PCR amplification of the gene from U251 cell cDNA using the following primers including the restriction sites for NheI and Hind III,

Fw: 5′GCTACGGCTAGCATGATTCCCTTTTTACCCATG′3,

Rv: 5′ GCTACGAAGCTTTCACTGAGAACGACCTTCCC′3.

For the Luciferase reporter assay a reporter construct with 1000bp of the POSTN promoter upstream of the transcription start site were cloned into the pGL3-Basic Vector (Promega). The region was amplified using PCR from genomic U251 cell DNA using the following primers including restriction sites for SacI and BgIII:

Fw: 5′CATGGAGCTCTGGCTAGGGATTGCATAGTGT′3,

Rv: 5′CATGAGATCTAGAACTGGCAGTGGGCTTTG′3.

A deletion mutant of the plasmid was created using the Quick Change II XL Site directed mutagenesis Kit (Agilent Technologies) and the following primers

Fw: 5′GATGTGCTGCATAGATTCAACATCAC3′,

Rv: 5′GTGATGTTGAATCTATGCAGCACATC3′.

Restriction enzymes and T4 DNA Ligase were purchased from NEB and restriction digest, ligation and transformation into competent cells (DH5α, Life Technologies) were carried out following the supplier's instructions.

### Inverted invasion assay

The inverted invasion assay was carried out as described previously [116]. In brief, geltrex (Gibco, A1413302) was mixed 1:1 with PBS and supplemented with 25 μg/ml fibronectin (FN, Sigma), 60 μl of the mix was added to each transwell dish (8 μm polycarbonate membrane, Costar) and allowed to set at 37°C for 45 min. Next 30,000 cells were added to the top of the membrane and transwells were incubated upside down for 4 h to allow cells to settle on the membrane. The wells were then washed twice by dipping into serum free media and placed in 1 ml serum free media in a 24 well plate. On top of the geltrex layer 100 μl serum-rich media was added supplemented with 10 ng/ml EGF and 10 ng/ml HGF. Cells were incubated for 72 h and stained using 4 μM Calcein-AM (Santa Cruz Biotechnology) in media for 1 h. Invading cells were quantified using confocal microscopy by taking pictures every 10 μm throughout the geltrex matrix.

### Migration assay

Migration assays were carried out using the xCELLigence system (ACEA Bioscience) following the supplier's instructions. Briefly, cells were harvested and washed in serum free media, and 20,000 cells were added to the upper chamber of the CIM plate, with the lower chamber filled with serum rich media. Cells were allowed to migrate for up to 60 h and the numbers of migrated cells read every 15 min.

### Immunofluorescence

Cells were transfected and incubated as indicated in the figure legends and then fixed using 3 % paraformaldehyde in PBS. After blocking and permeabilisation for 1 h (0.4 % Triton X-100, 1 % BSA in PBS), the 96 well plates were incubated with α-GFAP antibody (1:500) in 1 % BSA in PBS overnight at 4°C. After 3 washes (10 min each) in PBST the slides were incubated in secondary antibody (1:1000) for 30 min at 37°C, washed again twice with PBST and then incubated for 10 min in PBS containing Hoechst Staining (1:10000). Cells were visualised using the Cellomics (Thermo Fisher).

### Colony formation assay

For the colony formation assay U251 cells were transfected with si scr or si p73. 24 h post transfection 500 cells were seeded per well of a 6 well plate in duplicate. After 2 weeks cells colonies were stained using crystal violet. The experiment was carried in 3 independent biological replicates.

### Annexin V/PI

U251 cells were seeded in 6 well plates and transfected with 30 nM siRNA targeting p73 or a scrambled control, 24 h post transfection cells were treated with 50 μM Temozolomide for the times indicated. Apoptosis was measured using the Annexin V-FITC Apoptosis Detection Kit (eBioscience) following the supplier's instructions. Staining was analysed using the FACS Calibur (Becton Dickinson) with CellQuestPro Software, per sample 10,000 cells were analysed.

### RT-qPCR and PCR

RNA was isolated from cells using the TRIZOL reagent (Life Technologies). For RT-qPCR first 3 μg of isolated RNA was digested using DNase (SIGMA) to eliminate any DNA contamination and then cDNA was generated using the Revert Aid cDNA synthesis kit (Life Technologies). Actual qPCR Primer for RT-qPCR were:

L32 (internal control) Fw: TTCCTGGTCCACAACGTCAAG

Rv: TGTGAGCGATCTCGGCAC,

total p73: Fw: GCCTGGAGCTGATGGAGTT,

Rv:ACGGGGGCTGTAGGTGAC,

POSTN: Fw:CTCATAGTCGTATCAGGGGTCG

Rv: ACACAGTCGTTTTCTGTCCAC.

RT-PCR and PCR were carried out as described previously [117].

### Microarray analysis

RNA was extracted from U251 cells that had been transfected with siRNA (scr or p73) 72 h prior to RNA isolation using TRIZOL (Invitrogen). Next, RNA was reverse transcribed, converted to cRNA, amplified, and labeled with a cyanine-3 dye using a Low Input Quick Amp labeling kit (Agilent). Then cRNAs were hybridized to human gene expression microarrays (Agilent, catalog number G4851B) containing 50,599 different probes. Slides were washed with the Agilent wash buffer reagents and scanned using the G2505C Agilent Microarray Scanner (scan control version A.8.4.1). Using the Agilent Feature Extraction software (version 10.7.3.1) data was extracted and analyzed using Agilent GeneSpring GX software (version 12.1). To analyze significant differential expression, an unpaired Student's t test with Benjamini-Hochberg multiple testing correction was applied. Hits with a *p*-value ≤ 0.05 and a fold change >2 were considered to be both statistically and biologically significant.

### Analysis of promoter region

To analyse potential p73 binding sites within the POSTN promoter an analysis was carried out using Math-Inspector Professional software and the TRANSFAC database on a region of 3000 kb upstream of the transcription-start site and within the first intron of human POSTN. The analysis highlighted a 25 bp region containing a p53-like RE site roughly 600 bp upstream of the transcription start site [118].

### Chromatin immunoprecipitation (ChIP) assay

ChIP assays were carried out using the MAGnify™ Chromatin Immunoprecipitation System (Life Technologies) following the manufacturer's instructions. Briefly, U251 cells were transfected with HA-TAp73α DNA, harvested 24 h post transfection and fixed using formaldehyde. After sonication an immunoprecipitation for TAp73 was performed using an HA antibody (Covance) or an IgG control, cross-linking was reversed and DNA eluted using the components of the kit. An PCR was performed on the input and diluted DNA using the following primers:

POSTN Fw:5′CAATATTGGCTGCTTTTCACCA′3,

Rv: 5′AAGGTTTGAAATGAAGCAGAAAGG′3,

MDM2

Fw: 5′GGTTGACTCAGCTTTTCCTCTTG′3,

Rv: 5′GGAAAATGCATGGTTTAAATAGCC′3.

### Bioinformatics analyses

Gene expression data sets GSE18166, GSE17538, GSE4573 and GSE19783 were downloaded from the GEO omnibus repository. The gene expression rank that reflects relative mRNA expression levels is more consistent as it does not require normalization and thus no normalization bias is introduced [94, 119–121]. Gene expression values were transformed into rank expression values on a scale from 100 to 0, where a rank value of 55 shows that 55% of probes in the sample have a lower expression value for a given gene. Pearson coefficient was used as a measure of correlation between expression profiles.

To divide the data sets into two cohorts, one where the positive correlation between expression profiles of total p73 and POSTN is maximized (in cohort 1) and one where it is minimized (cohort 2), first all samples are placed in cohort 1 and no samples in cohort 2. Next correlation between p73 and POSTN expression profiles in cohort 1 was computed, as well as the changes in correlation if one sample from the cohort was removed. The sample with maximal effect on correlation (maximal increase in positive correlation) is then moved from cohort 1 to cohort 2. This procedure is repeated until there is no sample left in cohort 1 which increases the positive correlation between p73 and POSTN, if it was removed. Next the statistical difference in survival of the two cohorts was calculated and graphs were blotted using R (statistical package).

## SUPPLEMENTARY MATERIAL FIGURES AND TABLE



## Abbreviations

POSTN: periostin
GBM: glioblastoma multiforme
TAp73: transactivation domain-containing p73 isoform
ΔNp73: amino-truncated p73 isoform
GFAP: glial fibrillary acidic protein
EMT: epithelial to mesenchymal transition
ChIP: chromatin immunoprecipitation.
