# Supplementary material for: p73 promotes glioblastoma cell invasion by directly activating POSTN (periostin) expression
Source: Oncotarget. 2016 Feb 22;7(11):11785–802. doi: 10.18632/oncotarget.7600 (PMC4914248; doi:10.18632/oncotarget.7600)
Supplement: Supplementary file 1 [file oncotarget-07-11785-s001.pdf]

p73 promotes glioblastoma cell invasion by directly activating POSTN (periostin) expression

Supplementary Material
p73 promotes glioblastoma cell invasion by directly activating POSTN (periostin) expression
Landré *et al*.

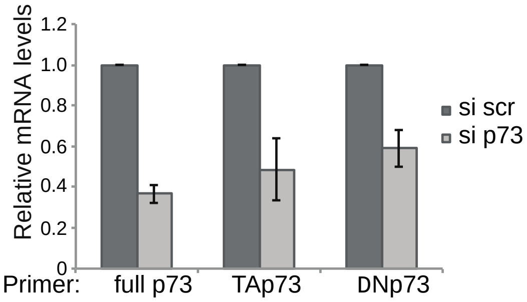

Figure S1 Knock down of full p73
RNA was extracted from U251 cells that were transfected with siRNA against full p73 and levels of mRNA of full, TAp73 and ΔNp73 was determined using RT-qPCR.

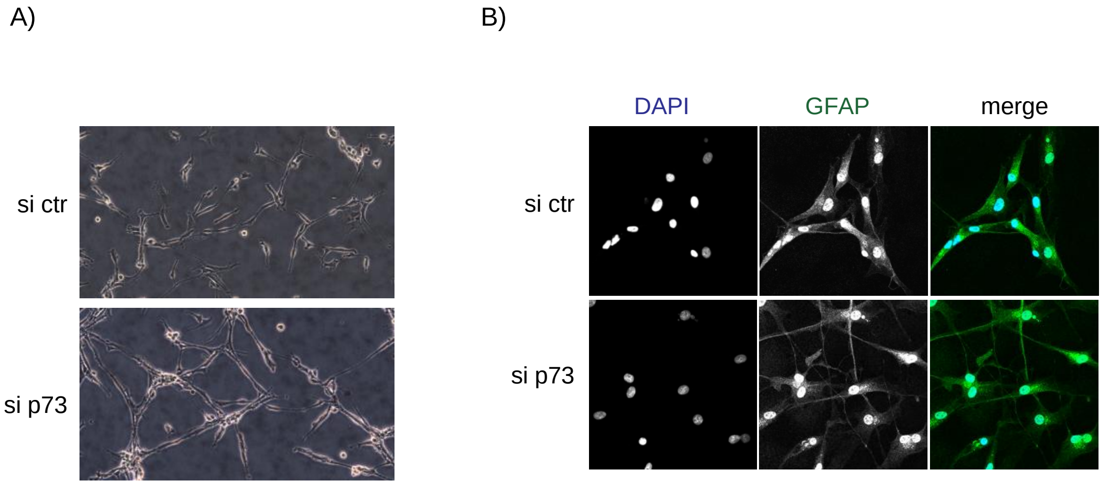

Figure S2 P73 knock down induces morphological transformation of U87 cells

A. Morphological changes of U87 cells after 72 h of p73 knock down using siRNA transfection. **B**. Cells as in B but fixed and stained with an anti-GFAP antibody.

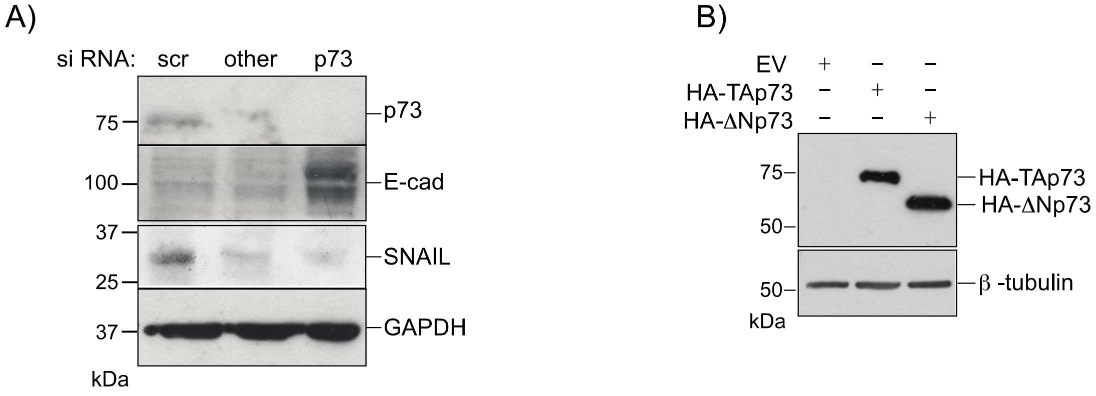

Figure S3. Overexpression and knock down of TAp73 and ΔNp73 in U251 cells.

A. Full scan of Figure 2A. Whole protein extract of U251 cells 72 h post-transfection with scr or p73 siRNA was analysed by immunoblotting with antibodies against p73, SNAIL, E-cadherin and GAPDH. **B.** Cells that were transfected with DNA for HA-TAp73α or HA-ΔNp73α (Figure 2D) were lysed and total protein extract was analysed using immunoblotting with antibodies detecting HA and β-tubulin as indicated.

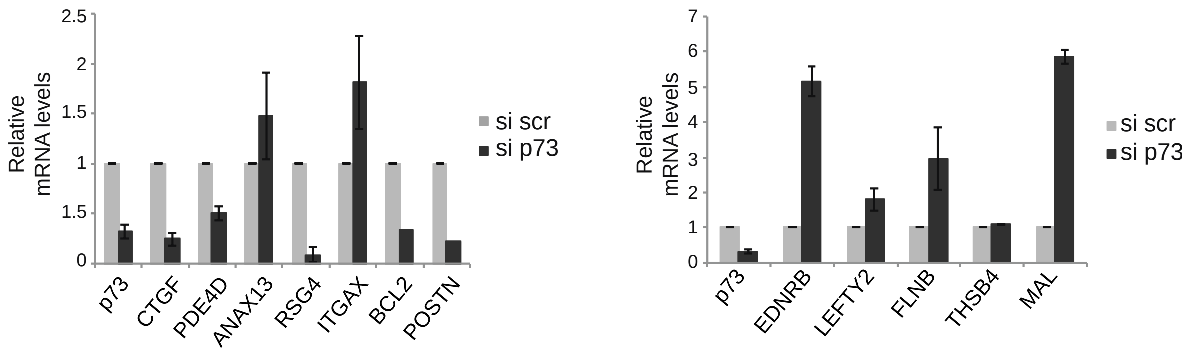

Figure S4. Validation of microarray.
Seven targets that were down regulated in the microarray after p73 knock down (left panel) and five targets that were up-regulated (right panel) were validated using RT-qPCR (mean of three independent experiments is shown).

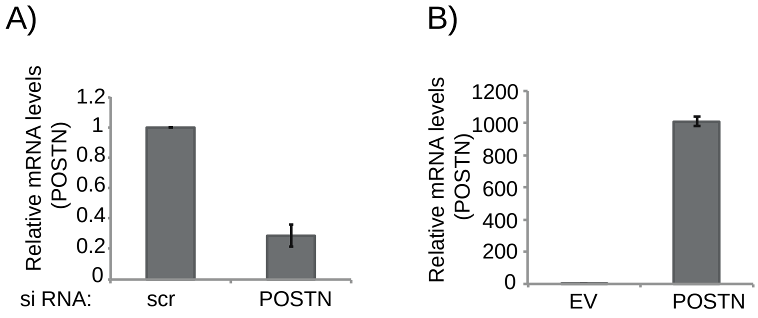

Figure S5. Knock-down and overexpression of POSTN in U251 cells. RNA was extracted from U251 cells that were transfected with either **(A)** siRNA for POSTN (or scr control) or **(B)** DNA encoding POSTN and RT-qPCR was performed to quantify POSTN mRNA levels.

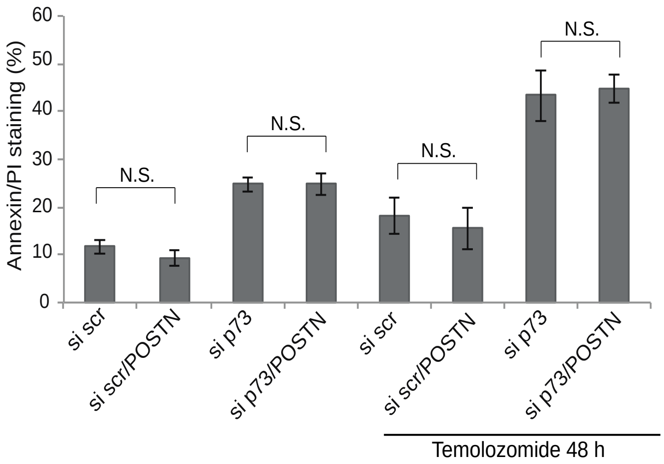

Figure S6. Effect POSTN overexpression on cell death after temozolomide treatment

U251 cells were transfected with siRNA (scr or p73) for 72 h and EV or POSTN for 24 h as indicated and then treated with 50 μM temozolomide for 48 h. Early and late apoptosis was detected using Annexin V/PI-double staining followed by flow cytometry analysis.

Table S1: RT-qPCR Primers used in Figure S2

Gene	Forward 3'→ 5'	Reverse 3'→ 5'
CTGF	CAGCATGGACGTTCGTCTG	AACCACGGTTTGGTCCTTGG
PDE4D	ACGGACCGGATAATGGAGGAG	ATTTTTCCACGGAAGCATTGTG
ANAX13	ACTTCGAGAAGACAGCGTTGG	GGACGGACTCATCTGTGCC
RSG4	ACATCGGCTAGGTTTCCTGC	GTTGTGGGAAGAATTGTGTTCAC
ITGAX	GGGATGCCGCCAAAATTCTC	ATTGCATAGCGGATGATGCCT
BCL2	GGTGGGGTCATGTGTGTGG	CGGTTCAGGTACTCAGTCATCC
LEFTY2	CAAGCTGGTCCGCTTTGC	TTGGTGCTTCAGGGTCACAG
FLNB	AACTGGCAAGACGGCAAAG	CGTGCATTATCCACAGGCTTC
THSB4	TGCTGCCAGTCCTGACAGA	GTTTAAGCGTCCCATCACAGTA
MAL	TCACCTTGGACGCAGCCTA	GAAGCCGTCTTGCATCGTGAT
EDNRB	TGCTGGGGATCATCGGGAA	GCGATCAAGATATTGGGACCGT
